# Anifrolumab Attenuates Follicular Helper T Cell Activation in Patients with Systemic Lupus Erythematosus

**DOI:** 10.3390/ijms26157397

**Published:** 2025-07-31

**Authors:** Ádám Diós, Ágnes Gyetvai, Gábor Papp, Tünde Tarr

**Affiliations:** Division of Clinical Immunology, Institute of Internal Medicine, Faculty of Medicine, University of Debrecen, H-4032 Debrecen, Hungary; dios.adam@med.unideb.hu (Á.D.); gyetvaia@med.unideb.hu (Á.G.); tarr.tunde@med.unideb.hu (T.T.)

**Keywords:** systemic lupus erythematosus, anifrolumab, follicular helper T cells

## Abstract

Systemic lupus erythematosus (SLE) is a severe autoimmune disease characterized by autoantibody production and multi-organ involvement. Anifrolumab, a monoclonal antibody targeting the type I interferon (IFN) receptor, has been approved for the treatment of SLE. Our aim was to investigate the long-term effects of inhibited type I IFN signaling on circulating follicular helper T subsets (T_FH_), follicular regulatory T cells (T_FR_), and B lymphocyte subpopulations, reflecting the ongoing germinal center reactions in SLE patients. Peripheral blood samples were obtained from ten SLE patients before the initiation of anifrolumab treatment, and at months 6 and 12 of the intervention period. Flow cytometry analysis was performed to assess the frequencies of circulating T_FH_ cell subsets, T_FR_ cells, and certain B cell subpopulations. Serological parameters, including autoantibody levels and complement components, were determined as part of the routine diagnostic evaluation. We observed a significant and sustained reduction in the percentage of activated circulating T_FH_ cells. Notably, the frequency of CXCR3^−^CCR6^+^ T_FH_17 cells decreased, whereas the proportion of CXCR3^+^CCR6^−^ T_FH_1 cells increased significantly. Furthermore, the proportion of the IgD^−^CD27^−^ double-negative B lymphocytes was also significantly reduced. These findings suggest that anifrolumab therapy attenuates T_FH_ cell activation, which may contribute to its clinical efficacy by modulating germinal center responses in SLE.

## 1. Introduction

Systemic lupus erythematosus (SLE) is a chronic systemic autoimmune disorder with the potential to affect multiple organ systems, resulting in highly heterogeneous clinical manifestations [1]. Common symptoms include rash (typically the butterfly-shaped malar rash), fever, fatigue, arthritis, hematologic abnormalities, serositis, and neuropsychiatric manifestations, while life-threatening complications involve cardiovascular diseases and lupus nephritis [2]. The disease course is characterized by alternating periods of flare and remission.

The autoimmune response targets multiple nuclear autoantigens including double-stranded DNA (dsDNA), the Smith antigen (Sm), nucleosomes, U1-ribonucleoprotein (U1-RNP), and the SS-A (Ro) and SS-B (La) antigens. Autoantibodies play a central role in disease development, and the deposition of immune complexes can trigger organ inflammation. Germinal centers (GCs) within lymphoid follicles serve as key sites for B cell activation, clonal selection, and differentiation into memory B cells and antibody-producing plasma cells [3]. Two specialized CD4^+^ T cell subsets orchestrate the GC reaction: follicular helper T cells (T_FH_), which facilitate B cell activation and antibody production [4], and follicular regulatory T cells (T_FR_), which suppress excessive B cell responses [5]. In human studies, direct sampling of the lymph node is not feasible; thus, circulating peripheral T_FH_ and T_FR_ cells—considered memory counterparts of their GC-resident forms—are used as surrogate markers of GC activity [6]. These cells typically downregulate Bcl-6 expression while maintaining CXCR5 expression, allowing them to recirculate and re-enter lymphoid follicles, where they can resume their effector functions [7]. T_FH_ and T_FR_ cells have been implicated in the pathogenesis of SLE [8]. Elevated frequencies of PD-1^+^ICOS^+^ T_FH_ cells have been reported in SLE [9], and their levels correlate positively with disease activity scores [10], anti-dsDNA levels, and plasmablast counts [11]. Similar alterations in T_FH_ cells have also been described in primary Sjögren’s syndrome [12] and rheumatoid arthritis [13]. Subset analyses have revealed an increase in T_FH_17 and a decrease in T_FH_1 cell frequencies in SLE, a pattern also noted in other autoimmune conditions [14,15,16]. Although the suppressive functions of T_FR_ cells appear preserved in SLE, their frequencies are reduced [17,18]. These changes promote an enhanced GC reaction supporting B cell maturation and antibody production.

Classical immunosuppressive therapies have improved the life expectancy of patients with SLE; however, the side effects of glucocorticoids and the cumulative steroid dose have become leading contributors to complications and mortality [19]. Therefore, a major goal of current drug development is to enable steroid-sparing strategies. The introduction of monoclonal antibody therapies has significantly advanced the treatment of SLE by providing effective disease control with fewer adverse effects. Rituximab, a CD20-targeting antibody originally approved for rheumatoid arthritis, has been used off-label in SLE with promising results [20]. In 2011, belimumab—an antibody targeting the B lymphocyte stimulator (BlyS)—was the first biologic approved specifically for SLE [21]. More recently, in 2021, the FDA approved anifrolumab, a monoclonal antibody against interferon alpha/beta receptor subunit 1 (IFNAR1). Anifrolumab blocks type I interferon (IFN-I) signaling by inhibiting the Janus kinase (JAK)-signal transducer and activator of transcription (STAT) pathway, thereby suppressing IFN-stimulated gene expression and promoting receptor internalization [22]. Clinical trials have demonstrated its efficacy in improving the SLE Responder Index (SRI) and BILAG-based Composite Lupus Assessment (BICLA), reducing disease flares, alleviating skin and joint symptoms, and enabling a gradual decrease in the corticosteroid dose [23].

Type I interferons are key mediators of SLE pathogenesis, produced by various cell types, such as fibroblasts, epithelial cells, monocytes, macrophages, neutrophils, and plasmacytoid dendritic cells (pDCs) [24,25]. In SLE, excessive cell death and inflammation lead to the release of nucleic acids, which activate Toll-like receptors 7 and 9 (TLR7 and TLR9), triggering robust IFN-I production [25]. These cytokines promote dendritic cell maturation, upregulate MHC class II and co-stimulatory molecules, and stimulate T and B cell activation. Furthermore, type I IFNs stimulate the production of a variety of pro-inflammatory cytokines including interleukin-6 (IL-6), interleukin-15 (IL-15), granulocyte-macrophage colony-stimulating factor (GM-CSF), and BlyS [26].

Although IFN-I blockade is known to reduce inflammation in peripheral tissues [27], its potential effects on lymphoid follicular responses, particularly T_FH_ and T_FR_ cell dynamics, have not been thoroughly explored. It was reported that type I interferons promote IL-21 production, germinal center formation, and T_FH_ cell development [28,29]; hence, it may be hypothesized that the inhibition of type I IFN signaling might attenuate T_FH_ cell activation and suppress B cell stimulation. In this study, we examined the impact of anifrolumab therapy on circulating follicular T cell subsets and B cell subpopulations in patients with SLE.

## 2. Results

At the end of the 12-month follow-up period, we observed significant remission of the patients’ symptoms. The distributions of the clinical symptoms at baseline vs. after 12 months were as follows: arthritis *n* = 10 vs. *n* = 0; inflammatory-type rash *n* = 10 vs. *n* = 0, and oral or nasal mucosal ulcers *n* = 1 vs. *n* = 0. The SLE Disease Activity Index 2000 (SLE DAI 2K) showed a remarkable reduction (median: 9 [interquartile range (Q1–Q3): 8–10] before vs. 2 [1–3] at 12 months) (*p* = 0.002), while the Systemic Lupus International Collaborating Clinics (SLICC) Damage Index (mean ± standard deviation: 0.4 ± 0.7) did not change.

**Figure 1:**
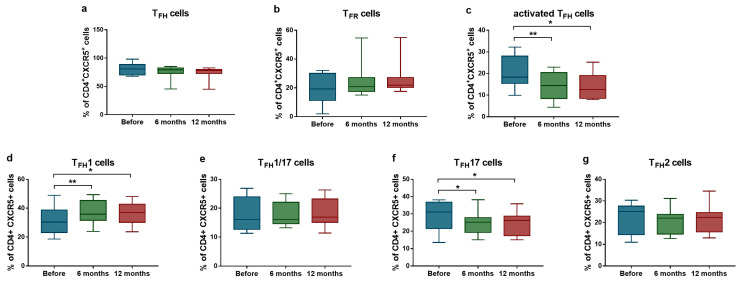
Ratios of activated T_FH_ cells and T_FR_ cells in SLE patients (*n* = 10) receiving anifrolumab therapy. Frequency of T_FH_ cells (**a**), T_FR_ cells (**b**), activated T_FH_ cells (**c**), T_FH_1 cells (**d**), T_FH_1/17 cells (**e**), T_FH_17 cells (**f**) and T_FH_2 cells (**g**) were evaluated by flow cytometric analysis. Boxes represent interquartile ranges, horizontal lines show median values, and whiskers show minimum and maximum values. Statistically significant differences are indicated by * *p* < 0.05; ** *p* < 0.01.

Considering the laboratory findings, a trend was observed toward a decreased proportion of T_FH_ cells (median: 80.8% [Q1–Q3: 70.0–88.7%] vs. median: 78.2% [Q1–Q3: 72.9–79.7%]; *p* = 0.098 after 12 months) and an increased proportion of T_FR_ cells (median: 19.2% [Q1–Q3: 11.3–30.1%] vs. median: 21.8% [Q1–Q3: 20.3–27.2%]; *p* = 0.098 after 12 months), both approaching statistical significance (Figure 1a,b). We found significant reduction in the proportion of PD1^+^ICOS^+^ activated T_FH_ cell population (median: 18.3% [Q1–Q3: 15.4–18.3%] vs. median: 14.4% [Q1–Q3: 8.5–20.4%]; *p* = 0.004 after 6 months; and median: 18.3% [Q1–Q3: 15.4–18.3%] vs. median: 12.6% [Q1–Q3: 8.5–19.1%]; p = 0.039 after 12 months) (Figure 1c). Analysis of T_FH_ subpopulations revealed a significant increase in the ratio of T_FH_1 cells (median: 30.3% [Q1–Q3: 23.1–38.7%] vs. median: 35.8% [Q1–Q3: 31.6–45.3%]; *p* = 0.0049 after 6 months; and median: 30.3% [Q1–Q3: 23.1–38.7%] vs. median: 36.9% [Q1–Q3: 30.1–42.8%] *p* = 0.015 after 12 months), while the proportion of T_FH_17 cells significantly decreased (median: 31.0% [Q1–Q3: 21.6–36.8%] vs. median: 25.2% [Q1–Q3: 19.4–27.8%]; *p* = 0.037 after 6 months; and median: 31.0% [Q1–Q3: 21.6–36.8%] vs. median: 26.2% [Q1–Q3: 17.5–28.7%]; *p* = 0.038 after 12 months) (Figure 1d,f).

**Figure 2:**
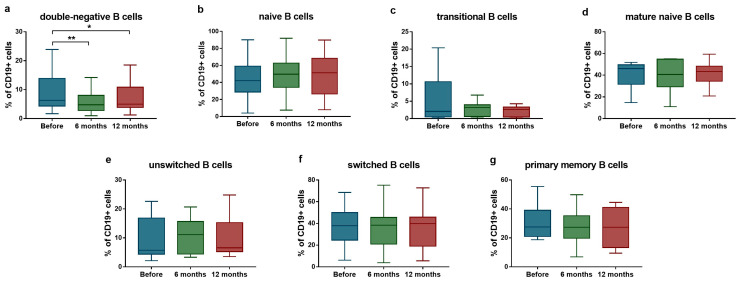
Ratio of B lymphocyte subsets in SLE patients (*n* = 10) upon anifrolumab therapy. Frequency of double-negative B cells (**a**), naive B cells (**b**), transitional B cells (**c**), mature naive B cells (**d**), unswitched B cells (**e**), switched B cells (**f**) and primary memory B cells (**g**) were evaluated by flow cytometric analysis. Boxes represent interquartile ranges, horizontal lines show median values, and whiskers show minimum and maximum values. Statistically significant differences are indicated by * *p* < 0.05; ** *p* < 0.01.

Regarding B cell subsets, a significant reduction was detected in the proportion of IgD^−^CD27^−^ double-negative B cells (median: 6.3% [Q1–Q3: 4.3–13.9%] vs. median: 4.8% [Q1–Q3: 2.8–8.0%]; *p* = 0.004 after 6 months; and median: 6.3% [Q1–Q3: 4.3–13.9%] vs. median: 5.0% [Q1–Q3: 3.9–10.9%]; *p* = 0.039 after 12 months) (Figure 2a). No significant changes were observed in other B cell populations during the follow-up period (Figure 2b–g).

Serological parameters, including autoantibody profiles, immune complex levels, and complement components were assessed as part of routine diagnostic evaluation. Complement levels remained stable, as did anti-dsDNA levels throughout the follow-up. Only a few of the patients showed positivity for the other autoantibodies tested (anti-SS-A *n* = 3, anti-SS-B *n* = 2, anti-RNP *n* = 3, anti-Sm *n* = 3), which remained stable. However, a decreasing trend in anti-nuclear antibody (ANA) titers was observed, which may suggest a reduction in overall autoantibody production (Table 1).

## 3. Discussion

In recent decades, biological therapy has become a frontier in SLE treatment, with newly approved monoclonal antibodies offering targeted therapeutic strategies characterized by minimal adverse effects [1]. Anifrolumab is the most recently approved pharmacological agent for SLE, specifically targeting the interferon receptor alpha/beta subunit 1 (IFNAR1) [22]. Type I interferons have received significant attention as potential druggable targets in SLE, with over ten therapeutic agents currently undergoing clinical trials aimed at modulating the interferon pathways, thereby reflecting the therapeutic promise of interferon-targeted biologics [25].

The concept of a high interferon signature was established in the past decade, with findings indicating that 50–75% of adult and up to 90% of pediatric patients diagnosed with SLE exhibit an elevated type I IFN-driven gene expression profile [30]. The release of type I IFNs is a key pathogenetic event in the pathophysiology of SLE. These cytokines enhance antigen presentation and co-stimulation, thereby facilitating the activation of adaptive immune cells and serving as a critical link between the innate and adaptive immunity.

In this study, we investigated the effect of anifrolumab treatment on the germinal center reaction mediated by follicular T lymphocytes. Although the interaction between follicular helper T cells and B cells occurs within the lymphatic nodes, circulating peripheral CXCR5^+^ follicular T cells serve as reliable surrogates of the ongoing germinal center reaction, representing the memory phase counterparts of the lymph node resident T_FH_ cells [7].

Increased frequency of PD1^+^ICOS^+^ T_FH_ cells was previously reported in SLE patients [9] and it was found to correlate with the disease activity index [10], plasmablast expansion, and anti-dsDNA antibody levels [11]. In response to anifrolumab treatment, we observed a significant decrease in the ratio of activated T_FH_ cells, suggesting a diminished B cell activation signal. Although the proportion of T_FR_ cells did not change significantly, most patients showed an increase in T_FR_ cell frequency paralleling the reduction in T_FH_ cells. These findings highlight the substantial impact of anifrolumab on the T_FH_ compartment, given that T_FH_ over-activation is a key driver of the humoral autoimmune response in SLE.

The distribution of T_FH_ cell subsets in SLE has shown increased T_FH_17 and decreased T_FH_1 cell frequencies [14]. Moreover, elevation of T_FH_17 cells has been positively correlated with SLE disease activity index [31]. In our cohort, anifrolumab therapy resulted in a significant decrease in T_FH_17 cell proportion and a significant increase in T_FH_1 cells. This shift may have functional consequences for B cell activation, as T_FH_17 cells are potent inducers of B cell differentiation and antibody production, whereas T_FH_1 cells are comparatively less effective [7]. These results suggest that anifrolumab-mediated modulation of T_FH_ subsets may contribute to reduced B cell activation in SLE.

The proportion of IgD^−^CD27^−^ double-negative B lymphocytes is typically elevated in SLE, associated with increased autoantibody production and disease activity index [32]. In our study, this cell population was significantly decreased in response to the anifrolumab therapy, supporting the hypothesis of attenuated B cell stimulation.

Although autoantibody levels remained stable throughout the 12-month follow-up, a downward trend in ANA-titers was noted. This observation may indicate that sustained suppression of T_FH_ cell activity could ultimately reduce the activation of new B cell clones and autoantibody generation over time. Further longitudinal studies are needed to confirm this hypothesis.

The strengths of our study include the well-controlled follow-up period during which patients’ regular medical treatment remained unchanged and the novel investigation of the immunologic effects of anifrolumab therapy in patients. Limitations include the relatively small sample size and the absence of an age- and sex-matched SLE control group not receiving the anifrolumab therapy. Nonetheless, the self-controlled study design provides compelling internal consistency.

In summary, anifrolumab monoclonal antibody therapy may reduce follicular helper T cell activation and induce changes in the proportion of T_FH_ cell subsets that may attenuate B cell activation. Consequently, the blockage of type I IFN signaling may favorably influence the germinal center reaction and the T_FH_-B cell axis. Therefore, anifrolumab therapy may represent a sustainable treatment option for patients with SLE, potentially facilitating the gradual reduction of steroid use and promoting long-term remission.

## 4. Materials and Methods

### 4.1. Study Population

The study population consisted of ten patients with SLE (mean age: 45.00 ± 7.17 years). All patients were recruited from the outpatient clinic for systemic autoimmune diseases at the Division of Clinical Immunology, Institute of Internal Medicine, University of Debrecen, where they were undergoing regular follow-up care. The demographic characteristics, clinical features and ongoing treatment of patients are detailed in Table 2. Regarding the ongoing treatment of the patients, there were no changes in the administration of chloroquine, azathioprine, methotrexate, and mycophenolate mofetil; however, a reduction in the dosage of corticosteroids (5.85 ± 2.04 mg/day prior vs. 1.43 ± 1.90 mg/day after 12 months) was implemented.

During the study period, patients received 300 mg anifrolumab every 4 weeks. Blood samples were collected and laboratory tests were performed before the start of anifrolumab therapy, then after 6 and 12 months.

All SLE patients fulfilled the EULAR/American College of Rheumatology (ACR) 2019 classification criteria for lupus [33]. Chronic organ damage in SLE was determined using the SLICC Damage Index [34] and disease activity was quantified using SLEDAI-2K [35]. Participants with viral or bacterial infections as well as patients diagnosed with other chronic or autoimmune diseases were excluded. All procedures were approved by the Regional and Institutional Ethics Committee of the University of Debrecen (protocol number: 7019-2024). Informed written consent was obtained from all participants involved in this research, and the study was performed in accordance with the ethical standards of the Declaration of Helsinki.

### 4.2. Flow Cytometric Analysis

The flow cytometric analysis was performed as previously described [9]. Briefly, human PBMCs were isolated by density gradient centrifugation using Ficoll-Histopaque (Sigma-Aldrich, St Louis, MO, USA). The obtained cell suspension was labelled with the following fluorochrome-conjugated monoclonal antibodies: fluorescein isothiocyanate (FITC) anti-IgD (clone: IADB6, Beckman Coulter Inc., Fullerton, CA, USA), phycoerythrin (PE) anti-CD27 (clone: 1A4CD27, Beckman Coulter), phycoerythrin-Cyanine dye 5 (PE-Cy5) anti-CD19 (clone: J3-119, Beckman Coulter), FITC anti-CD38 (clone: HIT2, BioLegend, San Diego, CA, USA), allophycocyanin (APC) anti-CD24 (clone: ML5, BioLegend), Alexa Fluor 488 anti-CXCR5 (clone: RF8B2, BD Pharmingen, San Diego, CA, USA), PE anti-ICOS (clone: DX29, BD Pharmingen), Peridinin-chlorophyll protein-Cyanine dye 5.5 (PerCP-Cy5.5) anti-PD-1 (clone: EH12.1, BD Pharmingen), PE anti-CXCR3 (clone: G025H7, BioLegend), PerCP-Cy5.5 anti-CCR6 (clone: G034E3, BioLegend), PE anti-CD127 (clone: R34.34, Beckman Coulter), PE-Cy5 anti-CD25 (clone: B1.49.9, Beckman Coulter), and anti-CD4-APC (clone: RPA-T4, BioLegend). Cells were stained for 20 min at 4 °C in the dark, washed twice, and prepared for measurements. Multiparameter flow cytometry was performed using a FACS Calibur instrument (Becton Dickinson, Franklin Lakes, NJ, USA) and analyzed with FlowJo v10.0.7 software (Treestar, Ashland, OR, USA). In the case of B cells, at least 10,000 CD19^+^ events per sample were evaluated, while at least 50,000 CD4^+^ events per sample were recorded for circulating follicular T cells within the entire lymphocyte population. The proportions of B cell and follicular T cell subsets were analyzed within the total CD19^+^ B cells and CD4^+^CXCR5^+^ T cells, respectively.

**Figure 3:**
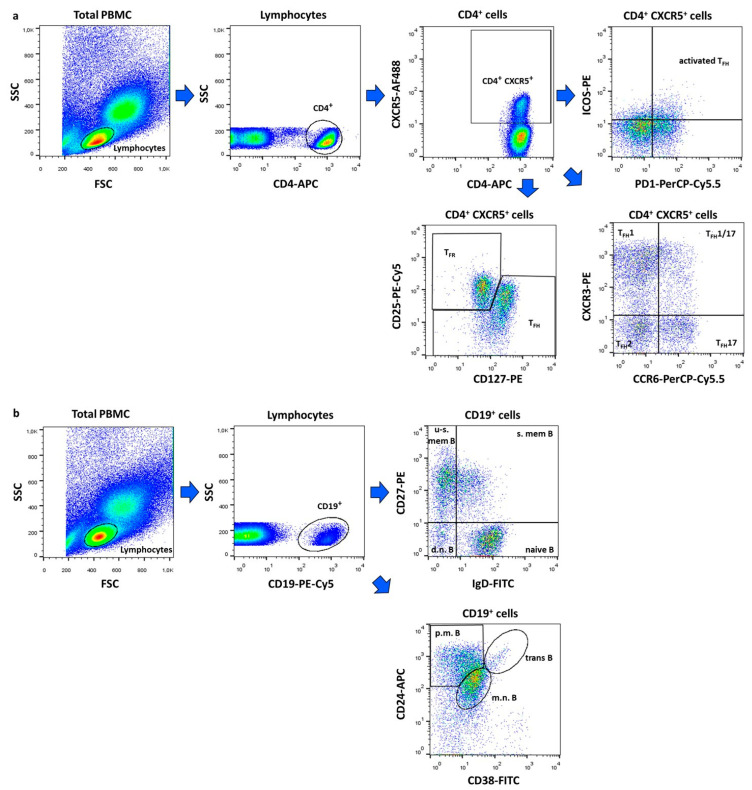
Gating strategy for flow cytometric immunophenotyping. Representative dot plots show the identification of follicular regulatory T (T_FR_) and follicular helper T (T_FH_) cell subtypes (**a**) and the characterization of B lymphocytes subsets (**b**). The following cell types were identified: activated T_FH_ (ICOS^+^PD1^+^), T_FH_1 (CXCR3^+^CCR6^−^), T_FH_1/17 (CXCR3^+^CCR6^+^), T_FH_2 (CXCR3^−^CCR6^−^) and T_FH_17 (CXCR3^−^CCR6^+^) and T_FR_ (CD25^+^CD127^−^) cells, d.n. = double negative (IgD^−^CD27^−^), naive (IgD^+^CD27^−^), u-s.mem. = un-switched memory (IgD^+^CD27^+^), s.mem. = switched memory (IgD^−^CD27^+^), m.n. = mature–naive (CD38^int^CD24^int^), p.m. = primarily memory (CD38^−^CD24^hi^), and trans = transitional (CD38^hi^CD24^hi^) B cells. Figures were exported from FlowJo v10.0.7 software.

Within CD4+CXCR5+ T cells, the following subsets were identified: activated T_FH_ (ICOS^+^PD1^+^), T_FH_1 (CXCR3^+^CCR6^−^), T_FH_1/17 (CXCR3^+^CCR6^+^), T_FH_2 (CXCR3^−^CCR6^−^) and T_FH_17 (CXCR3^−^CCR6^+^) and T_FR_ (CD25^+^CD127^−^) cells (Figure 3a). Among CD19^+^ B lymphocytes, we defined double negative (IgD^−^CD27^−^), naive (IgD^+^CD27^−^), un-switched memory (IgD^+^CD27^+^), switched memory (IgD^−^CD27^+^), mature–naive (CD38^int^CD24^int^), primarily memory (CD38^−^CD24^hi^), and transitional (CD38^hi^CD24^hi^) B cell subsets (Figure 3b).

### 4.3. Assessment of Humoral Immune Parameters

Immune serological parameters were determined from the serum samples. The presence of anti-nuclear antibodies (ANAs) was detected using an indirect immunofluorescence method on HEp2 cell line. Enzyme-linked immunosorbent assay (ELISA) was used for the detection of the following antibodies: anti-dsDNA (Orgentec, Mainz, Germany), anti-SS-A, anti-SS-B, anti-RNP, anti-Sm (Hycor, Biomedical, Garden Grove, CA, USA). Serum concentrations of complement C3 (normal range: 0.9 to 1.8 g/L) and C4 (normal range: 0.1 to 0.4 g/L) proteins were measured by a quantitative turbidimetric assay (Dialab GmbH, Wiener Neudorf, Austria). Immune complexes were detected by the polyethylene glycol precipitation method. All laboratory tests were performed under standardized conditions according to the manufacturer’s instructions, at the Department of Laboratory Medicine, Faculty of Medicine, University of Debrecen.

### 4.4. Statistical Analysis

Data and statistical analyses and graphic representation were performed using GraphPad Prism v7 software (GraphPad Software, San Diego, CA, USA). Descriptive data were represented as box plots of interquartile range (Q1–Q3) with a line in the middle as the median value. To assess the distribution of the data, the Shapiro–Wilk normality test was used. In case of normal distribution, we determined mean ± standard deviation (SD) values and used a two-tail paired t test. If the data set differed from normal distribution, we calculated median and Q1–Q3 and used the Wilcoxon test. Differences were considered statistically significant at *p* < 0.05.

## Figures and Tables

**Table 1:** Serological parameters of SLE patients upon anifrolumab therapy.

SLE Patients (*n* = 10)	Before	6 Months	12 Months
C3 g/L, median [Q1–Q3]	0.925 [0.625–1.183]	0.955 [0.838–1.185] *p* = 0.426	1.000 [0.868–1.208] *p* = 0.557
C4 g/L, median [Q1–Q3]	0.145 [0.090–0.170]	0.135 [0.085–0.195] *p* = 0.770	0.140 [0.095–0.233] *p* = 0.606
anti-dsDNA IU/mL, median [Q1–Q3]	26.5 [9.9–67.6]	25.5 [13.6–63.3] *p* = 0.594	20.7 [7.2–82.2] *p* = 0.770
ANA Hep2 titer, median [Q1–Q3]	1600 [320–3200]	640 [320–2560] *p* = 0.250	640 [320–1600] *p* = 0.063

C3—complement protein 3; C4—complement protein 4; ANA—anti-nuclear antibody staining of human epithelial cell line 2; Q1–Q3—interquartile range (25th–75th percentile). *p* < 0.05 was considered as statistically significant (numbers were always compared to the before values).

**Table 2:** Demographic and clinical features and treatments of SLE patients (*n* = 10).

	SLE Patients
Age in years, mean ± SD	45.00 ± 7.17
Female:Male ratio (*n*)	9:1
Disease duration in years, mean ± SD	13.22 ± 9.72
Lupus nephritis (*n*)	2
APS (*n*)	2
Methylprednisolone (*n*)	8
Chloroquine (*n*)	8
Azathioprine (*n*)	3
Methotrexate (*n*)	1
Mycophenolate mofetil (*n*)	1

APS—anti-phospholipid syndrome; SD—standard deviation.

## Data Availability

The original contributions presented in this study are included in the article; further inquiries can be directed to the corresponding author.
